# Exploiting vulnerabilities in cancer signalling networks to combat targeted therapy resistance

**DOI:** 10.1042/EBC20180016

**Published:** 2018-08-02

**Authors:** Peter T. Harrison, Paul H. Huang

**Affiliations:** Division of Molecular Pathology, The Institute of Cancer Research, London, U.K.

**Keywords:** Cancer Evolution, Combination therapies, Cell Signalling, Drug resistance, kinase inhibitors, Targeted therapy

## Abstract

Drug resistance remains one of the greatest challenges facing precision oncology today. Despite the vast array of resistance mechanisms that cancer cells employ to subvert the effects of targeted therapy, a deep understanding of cancer signalling networks has led to the development of novel strategies to tackle resistance both in the first-line and salvage therapy settings. In this review, we provide a brief overview of the major classes of resistance mechanisms to targeted therapy, including signalling reprogramming and tumour evolution; our discussion also focuses on the use of different forms of polytherapies (such as inhibitor combinations, multi-target kinase inhibitors and HSP90 inhibitors) as a means of combating resistance. The promise and challenges facing each of these polytherapies are elaborated with a perspective on how to effectively deploy such therapies in patients. We highlight efforts to harness computational approaches to predict effective polytherapies and the emerging view that exceptional responders may hold the key to better understanding drug resistance. This review underscores the importance of polytherapies as an effective means of targeting resistance signalling networks and achieving durable clinical responses in the era of personalised cancer medicine.

## Introduction

Deregulation of cellular signalling networks is a well-established driver of oncogenicity [1]. Mutations in proteins that comprise a number of key oncogenic signalling pathways have been identified in large-scale sequencing studies of tumour specimens [2]. These findings have spurred the development of targeted therapies that selectively inhibit these aberrant signalling proteins; and in some cancer types these drugs have shown significant clinical efficacy and patient benefit [3,4]. Despite initial tumour responses, there remains the major challenge of tumour relapse as nearly all patients ultimately develop resistance to such targeted therapies. In this review, we describe the major signalling mechanisms of resistance to targeted therapy. We outline the different approaches currently being explored to target signalling networks in order to overcome drug resistance, and conclude by offering a perspective on emerging approaches to achieve durable drug responses.

## Mechanisms of resistance

**Figure 1:**
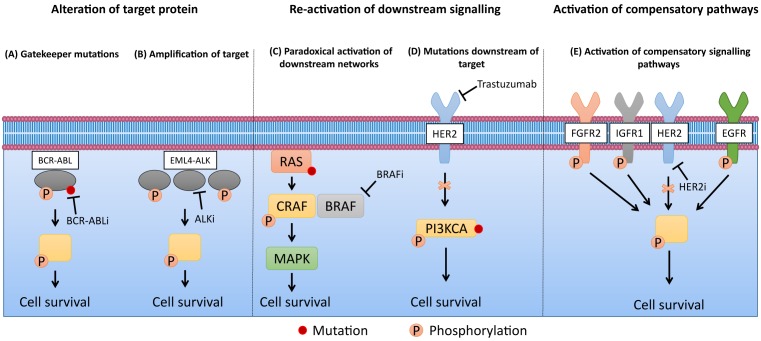
Different signalling mechanisms of drug resistance (**A**) Gatekeeper mutations on BCR–ABL restore kinase activity in the presence of BCR–ABL inhibitors (BCR–ABLi) in chronic myeloid leukaemia (ATP-binding site mutations and T315I enable resistance to first- and second-generation BCR–ABLi respectively) [86]. (**B**) Amplification of the EML4–ALK fusion restores signalling in the presence of ALK inhibitor (ALKi) crizotinib in NSCLC by increasing levels of active unbound oncogene [87]. (**C**) In the presence of oncogenic RAS, BRAF inhibitors (BRAFi) drive the formation of stable BRAF–CRAF complexes, resulting in hyperactivation of MAPK signalling in metastatic melanoma [12]. (**D**) PI3KCA mutations downstream of HER2 can re-activate signalling blocked by the anti-HER2 targeted agent trastuzumab, enabling resistance in HER2-positive breast cancer [88]. (**E**) Upregulation of multiple RTKs activates compensatory signalling pathways following HER2 inhibition in HER2-positive breast cancer [13].

Resistance to targeted therapies typically results from the re-activation of signalling pathways inhibited by the drug of interest. This can occur via (1) acquisition of drug resistant mutations or amplification of the target protein, (2) re-activation of downstream signalling proteins via paradoxical activation mechanisms or activating mutations, or (3) through activation of compensatory signalling pathways (Figure 1). A well-characterised example of drug-resistant mutations is found in the BCR–ABL fusion oncoprotein in chronic myeloid leukaemia. The kinase inhibitor imatinib binds to the ATP-binding site of BCR–ABL when it is in the closed conformation, resulting in kinase inactivation [5]. The most frequent mechanisms of resistance to imatinib are driven by mutations within the kinase domain which prevent BCR–ABL from adopting the conformation that enables high-affinity imatinib binding (e.g. E255K and M351T), and the T315I substitution which disrupts a hydrogen bond essential for imatinib binding [6] (Figure 1). Second generation BCR–ABL inhibitors dasatinib and nilotinib were developed to overcome imatinib resistance conferred by these mutations. Despite displaying increased potency compared with imatinib and clinical efficacy in many patients who have progressed on imatinib, these inhibitors are not effective against T315I substitutions [7–9]. A third generation inhibitor, Ponatinib, has since been advanced into the clinic and is effective in both wildtype and T315I mutant BCR–ABL [10]. Similar paradigms of second- and third-generation kinase inhibitors to tackle drug resistance mutations arising from first-generation inhibitors are also found in mutant EGFR and ALK-fusion positive non-small-cell lung cancer (NSCLC) [11]. A prominent example of resistance driven by re-activation of downstream signalling is through paradoxical activation of the MAPK (mitogen-activated protein kinase) pathway in BRAF V600E mutant melanoma. In the presence of oncogenic RAS, treatment with BRAF inhibitors (BRAFi) such as vemurafenib can drive the formation of BRAF–CRAF heterodimers, resulting in a stable complex that hyperactivates MAPK signalling leading to drug resistance [12]. The third major mechanism of drug resistance is through the activation of alternative compensatory signalling pathways, also known as “signalling reprogramming”. For example, it has been shown that transcriptional changes in response to the HER2 inhibitor lapatinib in HER2-positive breast cancer leads to the upregulation of multiple receptor tyrosine kinases (RTKs), including EGFR, IGF1R, INSR, FGFR2 and DDR2, which confer compensatory survival signalling and drug resistance [13]. Additional examples of other forms of signalling-based resistance mechanisms are provided in Figure 1.

Recent studies have shown that, in addition to cellular signalling mechanisms of resistance, an understanding of population-level evolutionary mechanisms is also necessary for developing effective strategies to overcome drug resistance [14,15]. Conceptually, it is now well-appreciated that there is significant intratumoural heterogeneity in patients, and that the selective pressure of drug treatment exacerbates evolutionary forces exerted on tumour cells, driving clonal selection and drug resistance. Work from Hata et al. [16] modelling the development of EGFR inhibitor (EGFRi) resistance in NSCLC *in vitro* has shown that acquisition of the EGFR gatekeeper mutation, T790M, can occur either through the accumulation of the mutation in drug-tolerant persister cells or through the selection of pre-existing clones which already possess the mutation. Evidence suggests that tumours evolve spatially within the primary tumour and at metastatic sites, as well as temporally during the course of disease and treatment. This is exemplified by reports of patients who harbour multiple resistant subclones with distinct mechanisms of drug resistance; a phenomenon termed polyclonal resistance [17,18]. In addition to these genetic-based mechanisms of drug resistance, transient changes to the transcriptome of individual cells can also lead to a stable drug-resistant state. Schaffer et al. [19] showed that addition of drug converts infrequent and transient transcriptional upregulation of resistance markers occurring in a small percentage of cells into stable transcriptional upregulation that promotes drug resistance.

Resistance to targeted therapy may occur through any combination of the mechanisms outlined above depending on the intratumoural heterogeneity at the time of treatment, the specific cancer type and the targeted therapy administered. Tumour-cell extrinsic mechanisms of resistance, such as the influence of the tumour microenvironment and the adaptive immune system, also operate in the context of targeted therapy. We do not discuss these mechanisms here, but they are reviewed elsewhere for readers who are interested [20,21]. Given that the common thread of targeted therapy resistance involves the re-activation of survival signalling pathways and the evolutionary selection of drug resistant clones, it may be possible to design strategies that selectively target these two processes with the ultimate goal of delaying or even preventing the onset of resistance. Here we focus on the use of polytherapies (i.e. therapies targeting multiple aspects of a cancer cell) to modulate signalling pathways and limit evolutionary selection as a means of achieving durable drug responses.

## Targeting signalling pathways to overcome resistance

### Combination therapy

Owing to the ability of tumour cells to circumvent blockade of an oncogene by a single therapeutic agent, there has been significant interest in identifying combination therapies using two or more drugs to enhance anti-tumour effects. By targeting multiple signalling pathways and resistant clones, combination therapies can delay the onset of resistance as they reduce the possible routes to re-activation of networks essential for tumour growth.

Combination therapies can be designed to target separate components of the same pathway to overcome re-activation of downstream signalling. An example is the combined use of MEK inhibitors (MEKi) with BRAFi in melanoma harbouring BRAF V600E mutations. Development of resistance to BRAFi in melanoma patients occurs at a median of 5 months post-treatment, with ∼80% of resistant tumours showing re-activation of the MAPK pathway [4,22,23]. Multiple mechanisms of resistance operate in this context. Acquisition of the p61 splice variant of BRAF-V600E promotes dimerization of BRAF, enabling ERK signalling in the presence of BRAFi [24]. Oncogenic mutations in RAS, such as G12, G13 and Q61 substitutions, can lead to the paradoxical activation of MAPK via stable BRAF–CRAF heterodimers which are formed following treatment with BRAFi [12]. Other less common mechanisms of resistance are acquisition of activating mutations in MEK and amplification of BRAF [23]. Independently, MEKi also improve overall survival in patients with melanoma harbouring BRAF V600E mutations compared with chemotherapy [25]. It was posited that combining the use of BRAFi and MEKi would delay the onset of resistance, as the combination would target the original driver oncogene and the pathway enabling secondary resistance. Preclinical models found that combination of BRAFi and MEKi delayed tumour relapse, and a phase III trial established a 25% relative reduction in the risk of disease progression in patients treated with the combination therapy compared to BRAFi monotherapy in a first line setting [26].

Alternatively, combination strategies can be designed to overcome resistance by simultaneously targeting multiple compensatory signalling pathways. Duncan et al. [27] showed that within 24 hours of MEKi treatment, triple negative breast cancer (TNBC) cells were able to re-activate ERK through the upregulation of multiple RTKs. The authors exploited this finding by utilising the multi-target RTK inhibitor sorafenib to sensitize TNBC cells to MEKi therapy. Further experiments in a genetically engineered mouse model of TNBC showed that combination of MEKi and sorafenib treatment achieved greater tumour regression compared with MEKi alone, while sorafenib monotherapy showed no effect. This demonstrates that targeting the initial driver oncogene in combination with pathways driving resistance in the first-line setting increases therapeutic efficacy.

One of the main challenges of combination therapies is defining *a priori* the best drug combinations to use (Table 1). Utilising molecular profiling approaches such as phosphoproteomics [28,29] to map signalling reprogramming associated with drug resistance and employing reverse combinatorial chemical [30] and genetic screens [31] to identify drug–genotype relationships can be very helpful in refining this process. Additionally, systems medicine strategies are being exploited to tailor drug combinations on a patient-specific basis. For instance, He et al. [32] developed a combined computational and experimental drug combination prediction and testing platform which integrates next-generation sequencing profiling with single-agent drug responses from both healthy cells and cancer cells obtained from patients to predict the most effective anti-cancer combinations. Another practical challenge with administering combination therapies is increased toxicity in patients. As well as overlapping toxicity profiles of different drugs, physicians must also consider novel toxicities specific to the combination treatment [33]. Finally, a significant obstacle to the effective use of combination therapies is the identification of predictive biomarkers. Biomarkers for targeted monotherapies are generally robust, as they typically involve alterations of the target gene (e.g. EGFR mutations or HER2 amplifications [34,35]). However, biomarker discovery may be more complex when considering combination strategies. For example, while BRAF and PIK3CA mutations are biomarkers for MEK inhibitors and AKT inhibitors respectively, neither are effective biomarkers for the combination of both classes of drugs [36,37]. Instead, KRAS mutations have been shown to be an effective biomarker for combination strategies of MEK and AKT inhibitors in patients [38]. The increasing use of genetically engineered mouse models and patient-derived xenograft models for preclinical testing of combination therapies may further improve the identification of biomarkers for these strategies [39,40].

**Table 1:** Advantages and disadvantages of combination therapies, multi-target kinase inhibitors (KIs), and HSP90 inhibitors as approaches for targeting networks to overcome resistance

	Combination therapy	Multi-target KI	HSP90 inhibitors
**Advantages**	• Selective inhibition of desired targets • Simultaneously targets initial driver oncogene and mechanism of resistance • Targets multiple subclones that are resistant to monotherapy	• Drug repurposing • Simultaneously targets initial driver oncogene and mechanism of resistance • Targets multiple subclones that are resistant to monotherapy • Predictable pharmacodynamics and pharmacokinetics • Targeting multiple kinases decreases the likelihood of resistance mechanisms occurring • Target tumour extrinsic factors such as angiogenesis, as well as tumour intrinsic factors	• Indirectly target many established oncogenes that are HSP90 client proteins • Low doses can limit genetic diversity which can prevent acquisition of drug resistance
**Disadvantages**	• Identification of efficacious combinations requires *a priori* knowledge of signaling networks • Unpredictable pharmacodynamics, pharmacokinetics and toxicity profiles • Lack of predictive biomarkers	• Identification of appropriate multi-target KIs requires *a priori* knowledge of signaling networks • Lack of predictive biomarkers • May have undesired effects on off-target kinases	• High toxicity at doses required for inhibition of client proteins • Potential risk of increasing genetic diversity in advanced cancers

### Multi-target kinase inhibitors

An alternative approach to overcome resistance is to exploit polypharmacology: the inhibition of multiple signalling targets using a single drug. Target-based development of selective inhibitors for kinases is the gold standard in drug discovery. However, due to the highly conserved architecture of the ATP-binding site in kinases, it has been challenging to develop inhibitors that are truly selective for a single kinase [41]. Indeed, screening efforts have revealed that drugs initially thought to be selective actually inhibit multiple off-target kinases [42,43]. In a recent demonstration of this phenomenon, Kuenzi et al. [44] used a screen of 240 compounds against 20 NSCLC cell lines and made the surprising discovery that the ALK inhibitor ceritinib showed potent activity against ALK-negative NSCLC lines. Chemical proteomics analysis found that ceritinib bound to multiple kinases including RSK1/2, FAK1, and IGF1R. Importantly, the authors determined that silencing of each of these three kinases individually did not induce significant cell death, indicating that ceritinib is exerting its anti-tumour effect through the simultaneous inhibition of multiple targets. Here we discuss the advantages of utilising polypharmacology and broad spectrum multi-target kinase inhibitors (KIs) as a means to target multiple signalling networks and overcome drug resistance.

One obvious advantage presented by multi-target KIs is the potential for drug repurposing. As we begin to understand the off-target effects of approved drugs, it is becoming possible to repurpose these molecules for new indications. A preclinical study of Ph+ acute lymphoblastic leukaemia demonstrated this utility. Zhao et al. [45] used a small-molecule drug screen to show that cells which were resistant to multiple BCR–ABL inhibitors developed collateral sensitivity to the multi-target KIs crizotinib, foretinib, vandetanib, and cabozantinib. Although these multi-target KIs all target MET, there was no overlap in sensitivity with other MET inhibitors – indicating an alternative mechanism to MET inhibition. A combination of phenotypic and signalling measurements revealed that these multi-target KIs exert their effect by targeting the BCR–ABL V299L mutation, which is only present in BCR–ABL inhibitor-resistant cells. As none of these drugs target wild type BCR–ABL, this study exemplifies how multi-target KIs can be readily repurposed for new indications.

**Figure 2:**
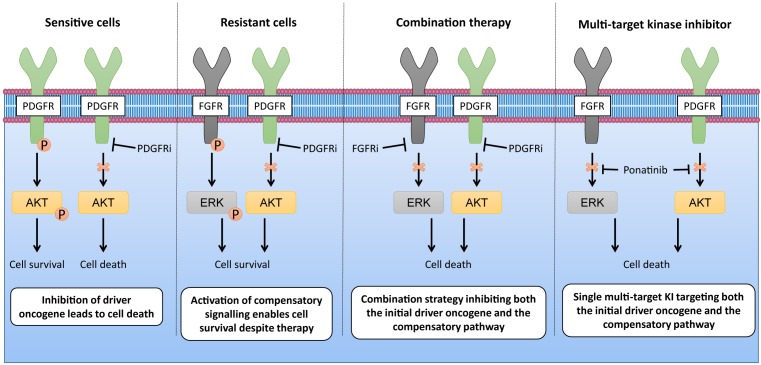
Combination therapies and multi-target KIs are able to overcome signalling re-activation and resistance This figure shows the effect of polytherapies on signalling in drug resistant cells. MRT cells develop resistance to PDGFR inhibitors (PDGFRi) by activating compensatory signalling pathways through upregulation of FGFR1. PDGFRi-resistant MRT cells are sensitive to polytherapies that target both FGFR1 and PDGFRA, either utilising a combination approach of PDGFRi and FGFR inhibitors (FGFRi) or a multi-target KI approach with a dual inhibitor.

Another advantage of utilising multi-target KIs is the ability to disrupt multiple resistance-causing signalling pathways simultaneously. The concept is to employ a single agent capable of targeting multiple signalling pathways in a cancer cell or multiple resistant subclones within a heterogeneous tumour to limit signalling re-activation and reprogramming in response to drug treatment, thus delaying or preventing the acquisition of resistance. A recent example of this is seen in PDGFRA inhibitor-resistant malignant rhabdoid tumours (MRTs). Wong et al. [46] showed that MRT cells that had acquired resistance to PDGFRA inhibitors showed elevated phosphorylation levels of the FGFR1 RTK. Correspondingly, dual treatment of MRT cells with PDGFRA and FGFR1 inhibitors increased apoptosis compared with treatment with each inhibitor alone, indicating a role for FGFR1 signalling in conferring resistance to PDGFRA inhibition (Figure 2). This led the authors to test the efficacy of ponatinib, a single agent that inhibits both FGFR1 and PDGFRA with equal potency. Ponatinib as a monotherapy was capable of overcoming PDGFRA inhibitor resistance and induced levels of apoptosis comparable to the combination of PDGFRA and FGFR1 inhibitors (Figure 2). This study indicates that multi-target KIs are capable of limiting the number of evolutionary options available to develop resistance by preventing both the initial driver event (e.g. PDGFRA) as well as the compensatory pathways which promote drug resistance (e.g. FGFR1).

Exploiting polypharmacology with multi-target KIs has a number of benefits over using combination-based therapies (Table 1). First, the use of a single drug with multiple targets is likely to have more predictable pharmacodynamics and pharmacokinetics than administering multiple single-target agents. Secondly, issues of adverse drug–drug interactions or antagonistic effects when using combination therapies are circumvented by using only one drug. Finally, targeting multiple processes in different cell types, including tumour-cell extrinsic factors such as angiogenesis, in addition to tumour-cell intrinsic kinase dependencies may further delay the onset of acquired resistance.

### HSP90 inhibitors

The HSP90 protein is an ATP-dependent molecular chaperone that is important for the folding, stabilisation and maturation of a wide range of “client proteins” [47]. In the context of cancer, many of these client proteins include well-established oncogenes such as mutant EGFR, EML4-ALK, BRAF and HER2 among others [48,49]. The discovery that HSP90 is essential for maintaining the stability and consequently functional activity of selected oncogenes led to the development of small-molecule HSP90 inhibitors with the premise that these compounds would function as broad-spectrum “super-kinase” inhibitors in oncology. Furthermore, given that multiple resistance-causing bypass pathways are driven by HSP90 client proteins, it was hypothesized that inhibiting this protein could prevent the development of secondary resistance commonly observed with KIs. While HSP90 inhibitors have shown remarkable efficacy in preclinical *in vitro* and *in vivo* studies in cancers such as mutant EGFR lung cancer [50–54], clinical trials of these inhibitors in NSCLC have unfortunately been disappointing with poor response rates and high toxicities in the first-line and acquired KI resistance settings [55–57].

Another perhaps less well-appreciated but central function of HSP90 is as a capacitor for phenotypic diversity [58]. HSP90 plays a key role in canalisation across phylogenetically diverse model organisms including flies, plants and fungi [58–60]. Canalisation is the ability of a population to maintain robust biological phenotypes despite perturbations in the environment or genotypic variation [61,62]. By regulating the folding of metastable genetic variants and allowing these proteins to perform wild-type biochemical functions, HSP90 limits phenotypic variation under basal conditions [63]. Canalisation allows for the accumulation of genetic diversity and heterogeneity within a population over time, which can lead to the emergence of previously silent genetic and signalling variants as new traits during selection by extreme environmental stresses that overcome the buffering capacity of HSP90 [64]. In the context of drug resistance, environmental stress in the form of therapy will select for the most resistant clones from a genetically diverse pool of cells maintained by HSP90. It has been shown that low-dose, non-toxic inhibition of HSP90 is capable of limiting the generation of genetic diversity within the population and preventing the acquisition of drug resistance [59,65,66].

Given that tumour heterogeneity and the selection of drug-resistant clones is a major issue in cancer therapy, Whitesell et al. [67] investigated if HSP90 inhibitors have utility in overcoming hormonal therapy resistance in breast cancer. By exposing cell-line models to the second generation HSP90 inhibitor ganetespib at low, non-toxic doses in combination with the anti-oestrogen tamoxifen, they were successfully able to delay the development of tamoxifen resistance. Since high-dose HSP90 inhibition in humans induces a range of side effects, including ocular toxicities, that limit its utility as an anti-cancer agent [55,56], these data suggest an alternative approach would be to administer low, non-toxic doses of HSP90 inhibitors – to regulate canalisation and reduce intratumour heterogeneity – in combination with anti-oestrogens to achieve durable responses in patients. One caveat of this approach that has been observed in model organisms [58–60] is that there is a potential risk in advanced cancers that partial inhibition of HSP90 function alone at sub-lethal doses may reveal accumulated genetic diversity within heterogeneous tumours which could hasten the development of new malignant phenotypes, including new resistance-driving signalling pathways. While promising, more research is necessary in order to better characterise the effects of low-dose HSP90 inhibition on tumour heterogeneity and its use in combination with targeted therapy agents.

## Future perspectives

### Computational biology as a tool for identifying effective polytherapies

Increasingly, computational and mathematical modelling techniques are being used to predict signalling rewiring in response to targeted therapy [68], identify synthetic lethal interactions [69], and predict dosing schedules and drug combinations capable of delaying resistance [70,71]. The ability to identify *a priori* synthetic lethal interactions and signalling rewiring trajectories, in tandem with the capacity to predict the most efficacious targeted combinations and dosing schedules, would provide valuable information that could facilitate more rapid personalisation of treatment strategies.

Identifying kinase dependencies in tumours and assigning the best inhibitors for targeting those kinases is essential for rational design of targeted polytherapies. There have been multiple efforts to develop computational approaches to integrate omics based measurements to predict kinase dependencies in cancer cells. Examples include the Kinase Addiction Ranker (KAR) [72] and Kinase inhibitor connectivity map (K-Map) [73]. KAR is a computational algorithm that integrates high-throughput drug-screening data, quantitative drug–kinase binding data, and transcriptomics data to predict kinase dependencies in cancer cells, while K-Map is a web-based program that connects a set of query kinases to quantitative KI selectivity profiles, including multi-target KIs. These two approaches were used in tandem by Ryall et al. [74] to delineate the different kinase dependencies in TNBC and show for the first time that the multi-target KI bosutinib was effective in killing the HCC1806 TNBC cell line. Further development of next-generation computational strategies that are not only capable of predicting kinase dependencies in a cancer cell, but also factor in the kinase dependencies of multiple clones within a heterogeneous tumour, would advance our ability to rationally design effective polytherapies to prevent the acquisition of drug resistance [75].

Computational biology has also demonstrated utility in identifying effective polytherapies to tackle intratumoural heterogeneity. In a recent example, Jonsson et al.[76] utilised computational modelling based on tumour population dynamics to design treatment strategies that optimally controlled tumour growth. Analysing tumours from a patient with EGFRi-resistant NSCLC, the authors discovered that the patient had polyclonal resistance driven by amplification of the original EGFR mutation, acquisition of a gatekeeper mutation on EGFR, acquisition of BRAF V600E mutation, and MET amplification. Computational modelling employed to determine the most effective treatment strategy revealed that combination therapies were only able to control certain subpopulations within the tumour, while other subpopulations would continue to grow. For instance, combination of EGFR and BRAF inhibitors would control subpopulations harbouring BRAF mutations, but allow subpopulations with MET amplification to continue to grow. To achieve optimal tumour growth control, their modelling showed that the most effective strategy was to sequentially alternate between combination therapies to target distinct subpopulations within the tumour. Importantly, the authors show that switching to a different combination therapy even when an overall tumour response is observed led to the greatest reduction in tumour cells. This concept contrasts with current treatment paradigms, which advocate switching therapy only after the acquisition of drug resistance and tumour relapse.

### Exceptional responders as models for understanding mechanisms of drug resistance

In spite of the prevalence of acquired resistance to targeted therapies, there have been a small number of individuals, known as “exceptional responders”, who show remarkable sensitivity and durable response to treatment [77–79]. The National Cancer Institute has launched the Exceptional Responder Initiative following a review of unpublished data from phase II clinical trials that identified exceptional responses in up to 10% of patients where the drug did not receive FDA approval for that indication [80]. Through studying these exceptional responders, it may be possible to uncover genotypes and mechanisms that confer long-term sensitivity to specific targeted agents.

Recent molecular studies into such exceptional responders have uncovered a number of distinct mechanisms for durable responses to signalling pathway inhibitors. In one example, Al-Ahmadie et al. [78] identified a RAD50 mutation in a patient who showed durable response to a combination of CHK1 inhibition and chemotherapy. Through a combination of genetic and functional analyses they demonstrated that the RAD50 mutation attenuates ATM signalling and in the context of DNA damage resulting from chemotherapy, this attenuated signalling led to an extreme sensitivity to CHK1 inhibition. In another example of unusual durable responses, follow-up data from a phase III study of 946 patients with gastrointestinal stromal tumours, which began in 2001, showed that imatinib was still effective in 13% of patients after 10 years of treatment [81]. However, the biological mechanisms underpinning this durable response remain elusive. Future studies in the field should focus on delineating the signalling mechanisms underpinning exceptional response, which may aid in the development of effective salvage therapies for patients who go on to develop secondary resistance to these drugs.

## Conclusion

Polytherapies represent a promising avenue for improving cancer survival outcomes by reducing the rates of tumour recurrence in patients. Our increased understanding of the mechanisms of drug resistance has enabled the rational design of effective combination therapies and multi-target KI strategies capable of simultaneously targeting both the initial oncogenic driver and secondary mechanisms of resistance. Upfront as well as salvage delivery of these polytherapies may delay or prevent drug resistance by limiting the evolutionary routes available to tumour cells. Given the plasticity of signalling networks in cancer cells, there is a risk that resistance to polytherapies may still develop in patients. This possibility highlights the need to improve on our current ability to predict tumour clonal dynamics and evolution in response to targeted therapy, and to gain a deeper understanding of the biology that underpins the emergence of resistant disease. We anticipate that an increased use of computational modelling approaches to forecast cancer clonal evolution and signalling adaptation will be essential for eliminating the risk of developing resistance to polytherapies [82,83]. While significant obstacles to the widespread use of polytherapies in the clinic remain, the recent approval of a number of combination KI therapies indicates that these challenges are not insurmountable. Identifying effective polytherapies and predictive biomarkers, as well as overcoming issues of toxicity are crucial bottlenecks that need to be addressed. Moving forward, we anticipate that the increasing deployment of integrated experimental and computational strategies to identify effective combinations and dosing schedules together with employing innovative adaptive clinical trial designs to evaluate these drugs in patients should usher in a new generation of clinically efficacious polytherapies for use in precision cancer medicine [84,85].

## Summary

- Resistance to targeted therapy can occur via multiple heterogeneous mechanisms. There is a common thread of re-activation of survival signalling pathways and the evolutionary selection of drug-resistant clones.
- Combination and multi-target kinase inhibitor-based strategies are promising approaches for overcoming or preventing acquisition of kinase inhibitor resistance, as they are capable of limiting both bypass or compensatory signalling networks and the evolutionary routes available to cancer cells.
- Low doses of HSP90 inhibitors can limit the genetic diversity within a tumour and thus delay resistance.
- Novel computational strategies are facilitating better and more accurate identification of efficacious combination strategies and optimal dosing schedules.
- Studying exceptional responders will increase our understanding of durable responses to cancer therapies and may reveal genotypes and mechanisms that elicit long-term response.

## Abbreviations

ALK: anaplastic lymphoma kinase
BCR–ABLi: BCR–ABL inhibitor
BRAFi: BRAF inhibitor
CHK1: checkpoint kinase 1
DDR2: discoidin domain-containing receptor 2
EGFRi: EGFR inhibitor
EGFR: epidermal growth factor receptor
FAC1: focal adhesion kinase 1
FGFR2: fibroblast growth factor receptor 2
HER2: erb-b2 receptor tyrosine kinase 2
HSP: heat shock protein
INSR: Insulin receptor
IGF1R: Insulin-like growth factor 1 receptor,
KAR: kinase addiction ranker
KI: kinase inhibitor
MAPK: mitogen-activated protein kinase
MEK: mitogen-activated protein kinase kinase
MEKi: MEK inhibitor
MRT: malignant rhabdoid tumour
NSCLC: non-small-cell lung cancer
PDGFRA: platelet-derived growth factor receptor alpha
PIK3CA: phophatidylinositol-4,5-bisphosphate 3-kinase catalytic subunit alpha
RAD50: RAD50 double strand break repair protein
RSK1/2: ribosomal S6 kinase
RTK: receptor tyrosine kinase
TNBC: triple negative breast cancer

## References

[1] Lemmon M.A. and Schlessinger J. (2010) Cell signaling by receptor tyrosine kinases. Cell 141, 1117–1134 10.1016/j.cell.2010.06.011 20602996 PMC2914105

[2] Leiserson M. D.M., Vandin F., Wu H.-T., Dobson J.R., Eldridge J.V, Thomas J.L. (2015) Pan-cancer network analysis identifies combinations of rare somatic mutations across pathways and protein complexes. Nat. Genet. 47, 106–114 10.1038/ng.3168 25501392 PMC4444046

[3] Lynch T.J., Bell D.W., Sordella R., Gurubhagavatula S., Okimoto R.A., Brannigan B.W. (2004) Activating Mutations in the Epidermal Growth Factor Receptor Underlying Responsiveness of Non–Small-Cell Lung Cancer to Gefitinib. N. Engl. J. Med. 350, 2129–2139 10.1056/NEJMoa040938 15118073

[4] Chapman P.B., Hauschild A., Robert C., Haanen J.B., Ascierto P., Larkin J. (2011) Improved Survival with Vemurafenib in Melanoma with BRAF V600E Mutation. N. Engl. J. Med. 364, 2507–2516 10.1056/NEJMoa1103782 21639808 PMC3549296

[5] Schindler T., Bornmann W., Pellicena P., Miller W.T., Clarkson B. and Kuriyan J. (2000) Structural mechanism for STI-571 inhibition of abelson tyrosine kinase. Science 289, 1938–1942 10.1126/science.289.5486.1938 10988075

[6] Shah N.P., Nicoll J.M., Nagar B., Gorre M.E., Paquette R.L., Kuriyan J. (2002) Multiple BCR-ABL kinase domain mutations confer polyclonal resistance to the tyrosine kinase inhibitor imatinib (STI571) in chronic phase and blast crisis chronic myeloid leukemia. Cancer Cell 2, 117–125 10.1016/S1535-6108(02)00096-X 12204532

[7] Kantarjian H., Shah N.P., Hochhaus A., Cortes J., Shah S., Ayala M. (2010) Dasatinib versus Imatinib in Newly Diagnosed Chronic-Phase Chronic Myeloid Leukemia. N. Engl. J. Med. 362, 2260–2270 10.1056/NEJMoa1002315 20525995

[8] Saglio G., Kim D.-W., Issaragrisil S., le Coutre P., Etienne G., Lobo C. (2010) Nilotinib versus Imatinib for Newly Diagnosed Chronic Myeloid Leukemia. N. Engl. J. Med. 362, 2251–2259 10.1056/NEJMoa0912614 20525993

[9] O’Hare T., Walters D.K., Stoffregen E.P., Jia T., Manley P.W., Mestan J. (2005) In vitro activity of Bcr-Abl inhibitors AMN107 and BMS-354825 against clinically relevant imatinib-resistant Abl kinase domain mutants. Cancer Res. 65, 4500–4505 10.1158/0008-5472.CAN-05-0259 15930265

[10] Huang W.-S., Metcalf C.A., Sundaramoorthi R., Wang Y., Zou D., Thomas R.M. (2010) Discovery of 3-[2-(imidazo[1,2-b]pyridazin-3-yl)ethynyl]-4-methyl-N-{4-[(4-methylpiperazin-1-yl)methyl]-3-(trifluoromethyl)phenyl}benzamide (AP24534), a potent, orally active pan-inhibitor of breakpoint cluster region-abelson (BCR-ABL) kinase including the T315I gatekeeper mutant. J. Med. Chem. 53, 4701–4719 10.1021/jm100395q 20513156

[11] Gainor J.F. and Shaw A.T. (2013) Emerging paradigms in the development of resistance to tyrosine kinase inhibitors in lung cancer. J. Clin. Oncol. 31, 3987–3996 10.1200/JCO.2012.45.2029 24101047 PMC3805932

[12] Heidorn S.J., Milagre C., Whittaker S., Nourry A., Niculescu-Duvas I., Dhomen N. (2010) Kinase-dead BRAF and oncogenic RAS cooperate to drive tumor progression through CRAF. Cell 140, 209–221 10.1016/j.cell.2009.12.040 20141835 PMC2872605

[13] Stuhlmiller T.J., Miller S.M., Zawistowski J.S., Nakamura K., Beltran A.S., Duncan J.S. (2015) Inhibition of Lapatinib-Induced Kinome Reprogramming in ERBB2-Positive Breast Cancer by Targeting BET Family Bromodomains. Cell Rep. 11, 390–404 10.1016/j.celrep.2015.03.037 25865888 PMC4408261

[14] Andre F., Mardis E., Salm M., Soria J.-C., Siu L.L. and Swanton C. (2014) Prioritizing targets for precision cancer medicine. Ann. Oncol. 25, 2295–2303 10.1093/annonc/mdu478 25344359

[15] Alizadeh A.A., Aranda V., Bardelli A., Blanpain C., Bock C., Borowski C. (2015) Toward understanding and exploiting tumor heterogeneity. Nat. Med. 21, 846–853 10.1038/nm.3915 26248267 PMC4785013

[16] Hata A.N., Niederst M.J., Archibald H.L., Gomez-Caraballo M., Siddiqui F.M., Mulvey H.E. (2016) Tumor cells can follow distinct evolutionary paths to become resistant to epidermal growth factor receptor inhibition. Nat. Med. 22, 262–269 10.1038/nm.4040 26828195 PMC4900892

[17] Choi Y.L., Soda M., Yamashita Y., Ueno T., Takashima J., Nakajima T. (2010) EML4-ALK Mutations in Lung Cancer That Confer Resistance to ALK Inhibitors. N. Engl. J. Med. 363, 1734–1739 10.1056/NEJMoa1007478 20979473

[18] Blakely C.M., Watkins T. B.K., Wu W., Gini B., Chabon J.J., McCoach C.E. (2017) Evolution and clinical impact of co-occurring genetic alterations in advanced-stage EGFR-mutant lung cancers. Nat. Genet. 49, 1693–1704 10.1038/ng.3990 29106415 PMC5709185

[19] Shaffer S.M., Dunagin M.C., Torborg S.R., Torre E.A., Emert B., Krepler C. (2017) Rare cell variability and drug-induced reprogramming as a mode of cancer drug resistance. Nature 546, 431–435 10.1038/nature22794 28607484 PMC5542814

[20] Sun Y. (2016) Tumor microenvironment and cancer therapy resistance. Cancer Lett. 380, 205–215 10.1016/j.canlet.2015.07.044 26272180

[21] Xu M.M., Pu Y., Zhang Y. and Fu Y.-X. (2016) The Role of Adaptive Immunity in the Efficacy of Targeted Cancer Therapies. Trends Immunol. 37, 141–153 10.1016/j.it.2015.12.007 26778079 PMC4738073

[22] Shi H., Hugo W., Kong X., Hong A., Koya R.C., Moriceau G. (2014) Acquired resistance and clonal evolution in melanoma during BRAF inhibitor therapy. Cancer Discov. 4, 80–93 10.1158/2159-8290.CD-13-0642 24265155 PMC3936420

[23] Rizos H., Menzies A.M., Pupo G.M., Carlino M.S., Fung C., Hyman J. (2014) BRAF inhibitor resistance mechanisms in metastatic melanoma: spectrum and clinical impact. Clin. Cancer Res. 20, 1965–1977 10.1158/1078-0432.CCR-13-3122 24463458

[24] Poulikakos P.I., Persaud Y., Janakiraman M., Kong X., Ng C., Moriceau G. (2011) RAF inhibitor resistance is mediated by dimerization of aberrantly spliced BRAF(V600E). Nature 480, 387–390 10.1038/nature10662 22113612 PMC3266695

[25] Flaherty K.T., Robert C., Hersey P., Nathan P., Garbe C., Milhem M. (2012) Improved Survival with MEK Inhibition in BRAF-Mutated Melanoma. N. Engl. J. Med. 367, 107–114 10.1056/NEJMoa1203421 22663011

[26] Long G.V., Stroyakovskiy D., Gogas H., Levchenko E., de Braud F., Larkin J. (2014) Combined BRAF and MEK Inhibition versus BRAF Inhibition Alone in Melanoma. N. Engl. J. Med. 371, 1877–1888 10.1056/NEJMoa1406037 25265492

[27] Duncan J.S., Whittle M.C., Nakamura K., Abell A.N., Midland A.A., Zawistowski J.S. (2012) Dynamic reprogramming of the kinome in response to targeted MEK inhibition in triple-negative breast cancer. Cell 149, 307–321 10.1016/j.cell.2012.02.053 22500798 PMC3328787

[28] Lima N., Lee A. T.J. and Huang P.H. (2017) Progress and impact of clinical phosphoproteomics on precision oncology. Transl. Cancer Res. 6, S1108–S1114 10.21037/tcr.2017.07.05

[29] Noujaim J., Payne L.S., Judson I., Jones R.L. and Huang P.H. (2016) Phosphoproteomics in translational research: a sarcoma perspective. Ann. Oncol. 27, 787–794 10.1093/annonc/mdw030 26802162

[30] Griner Mathews, A. L., Guha R., Shinn P., Young R.M., Keller J.M. (2014) High-throughput combinatorial screening identifies drugs that cooperate with ibrutinib to kill activated B-cell-like diffuse large B-cell lymphoma cells. Proc. Natl. Acad. Sci. U. S. A. 111, 2349–2354 10.1073/pnas.1311846111 24469833 PMC3926026

[31] Han K., Jeng E.E., Hess G.T., Morgens D.W., Li A. and Bassik M.C. (2017) Synergistic drug combinations for cancer identified in a CRISPR screen for pairwise genetic interactions. Nat. Biotechnol. 35, 463–474 10.1038/nbt.3834 28319085 PMC5557292

[32] He L., Tang J., Andersson E.I., Timonen S., Koschmieder S., Wennerberg K. (2018) Patient-Customized Drug Combination Prediction and Testing for T-cell Prolymphocytic Leukemia Patients. Cancer Res. 78, 2407–2418 10.1158/0008-5472.CAN-17-3644 29483097

[33] Park S.R., Davis M., Doroshow J.H. and Kummar S. (2013) Safety and feasibility of targeted agent combinations in solid tumours. Nat. Rev. Clin. Oncol. 10, 154–168 10.1038/nrclinonc.2012.245 23358316

[34] Advani P.P., Crozier J.A. and Perez E.A. (2015) HER2 testing and its predictive utility in anti-HER2 breast cancer therapy. Biomark. Med. 9, 35–49 10.2217/bmm.14.95 25605454

[35] Rosell R., Bivona T.G. and Karachaliou N. (2013) Genetics and biomarkers in personalisation of lung cancer treatment. Lancet 382, 720–731 10.1016/S0140-6736(13)61715-8 23972815

[36] Yap T.A., Bjerke L., Clarke P.A. and Workman P. (2015) Drugging PI3K in cancer: refining targets and therapeutic strategies. Curr. Opin. Pharmacol. 23, 98–107 10.1016/j.coph.2015.05.016 26117819 PMC4728196

[37] Poulikakos P.I., Zhang C., Bollag G., Shokat K.M. and Rosen N. (2010) RAF inhibitors transactivate RAF dimers and ERK signalling in cells with wild-type BRAF. Nature 464, 427–430 10.1038/nature08902 20179705 PMC3178447

[38] Tolcher A.W., Patnaik A., Papadopoulos K.P., Rasco D.W., Becerra C.R., Allred A.J. (2015) Phase I study of the MEK inhibitor trametinib in combination with the AKT inhibitor afuresertib in patients with solid tumors and multiple myeloma. Cancer Chemother. Pharmacol. 75, 183–189 10.1007/s00280-014-2615-5 25417902

[39] Robles A.I. and Varticovski L. (2008) Harnessing genetically engineered mouse models for preclinical testing. Chem. Biol. Interact. 171, 159–164 10.1016/j.cbi.2007.01.014 17362899

[40] Hidalgo M., Amant F., Biankin A.V, Budinská E., Byrne A.T., Caldas C. (2014) Patient-derived xenograft models: an emerging platform for translational cancer research. Cancer Discov. 4, 998–1013 10.1158/2159-8290.CD-14-0001 25185190 PMC4167608

[41] Zhang J., Yang P.L. and Gray N.S. (2009) Targeting cancer with small molecule kinase inhibitors. Nat. Rev. Cancer 9, 28–39 10.1038/nrc2559 19104514 PMC12406740

[42] Roth B.L., Sheffler D.J. and Kroeze W.K. (2004) Magic shotguns versus magic bullets: selectively non-selective drugs for mood disorders and schizophrenia. Nat. Rev. Drug Discov. 3, 353–359 10.1038/nrd1346 15060530

[43] Fabian M.A., Biggs W.H., Treiber D.K., Atteridge C.E., Azimioara M.D., Benedetti M.G. (2005) A small molecule–kinase interaction map for clinical kinase inhibitors. Nat. Biotechnol. 23, 329–336 10.1038/nbt1068 15711537

[44] Kuenzi B.M., Remsing Rix L.L., Stewart P.A., Fang B., Kinose F., Bryant A.T. (2017) Polypharmacology-based ceritinib repurposing using integrated functional proteomics. Nat. Chem. Biol. 13, 1222–1231 10.1038/nchembio.2489 28991240 PMC5909815

[45] Zhao B., Sedlak J.C., Srinivas R., Creixell P., Pritchard J.R., Tidor B. (2016) Exploiting Temporal Collateral Sensitivity in Tumor Clonal Evolution. Cell 165, 234–246 10.1016/j.cell.2016.01.045 26924578 PMC5152932

[46] Wong J.P., Todd J.R., Finetti M.A., McCarthy F., Broncel M., Vyse S. (2016) Dual Targeting of PDGFRα and FGFR1 Displays Synergistic Efficacy in Malignant Rhabdoid Tumors. Cell Rep. 17, 1265–1275 10.1016/j.celrep.2016.10.005 27783942 PMC5098123

[47] Barrott J.J. and Haystead T. A.Hsp90 (2013) an unlikely ally in the war on cancer. FEBS J. 280, 1381–1396 10.1111/febs.12147 23356585 PMC3815692

[48] Shimamura T., Lowell A.M., Engelman J.A. and Shapiro G.I. (2005) Epidermal growth factor receptors harboring kinase domain mutations associate with the heat shock protein 90 chaperone and are destabilized following exposure to geldanamycins. Cancer Res. 65, 6401–6408 10.1158/0008-5472.CAN-05-0933 16024644

[49] Trepel J., Mollapour M., Giaccone G. and Neckers L. (2010) Targeting the dynamic HSP90 complex in cancer. Nat. Rev. Cancer 10, 537–549 10.1038/nrc2887 20651736 PMC6778733

[50] Shimamura T., Li D., Ji H., Haringsma H.J., Liniker E., Borgman C.L. (2008) Hsp90 inhibition suppresses mutant EGFR-T790M signaling and overcomes kinase inhibitor resistance. Cancer Res. 68, 5827–5838 10.1158/0008-5472.CAN-07-5428 18632637 PMC3272303

[51] Sawai A., Chandarlapaty S., Greulich H., Gonen M., Ye Q., Arteaga C.L. (2008) Inhibition of Hsp90 down-regulates mutant epidermal growth factor receptor (EGFR) expression and sensitizes EGFR mutant tumors to paclitaxel. Cancer Res. 68, 589–596 10.1158/0008-5472.CAN-07-1570 18199556 PMC4011195

[52] Xu W., Soga S., Beebe K., Lee M.J., Kim Y.S., Trepel J. (2007) Sensitivity of epidermal growth factor receptor and ErbB2 exon 20 insertion mutants to Hsp90 inhibition. Br. J. Cancer 97, 741–744 10.1038/sj.bjc.6603950 17712310 PMC2360392

[53] Xu L., Kikuchi E., Xu C., Ebi H., Ercan D., Cheng K.A. (2012) Combined EGFR/MET or EGFR/HSP90 inhibition is effective in the treatment of lung cancers codriven by mutant EGFR containing T790M and MET. Cancer Res. 72, 3302–3311 10.1158/0008-5472.CAN-11-3720 22552292 PMC3389159

[54] Courtin A., Smyth T., Hearn K., Saini H.K., Thompson N.T., Lyons J.F. (2016) Emergence of resistance to tyrosine kinase inhibitors in non-small-cell lung cancer can be delayed by an upfront combination with the HSP90 inhibitor onalespib. Br. J. Cancer 115, 1069–1077 10.1038/bjc.2016.294 27673365 PMC5117788

[55] Sequist L.V, Gettinger S., Senzer N.N., Martins R.G., Janne P.A., Lilenbaum R. (2010) Activity of IPI-504, a novel heat-shock protein 90 inhibitor, in patients with molecularly defined non-small-cell lung cancer. J. Clin. Oncol. 28, 4953–4960 10.1200/JCO.2010.30.8338 20940188 PMC4676802

[56] Johnson M.L., Yu H.A., Hart E.M., Weitner B.B., Rademaker A.W., Patel J.D. (2015) Phase I/II Study of HSP90 Inhibitor AUY922 and Erlotinib for EGFR-Mutant Lung Cancer With Acquired Resistance to Epidermal Growth Factor Receptor Tyrosine Kinase Inhibitors. J. Clin. Oncol. 33, 1666–1673 10.1200/JCO.2014.59.7328 25870087 PMC4881377

[57] Socinski M.A., Goldman J., El-Hariry I., Koczywas M., Vukovic V., Horn L. (2013) A multicenter phase II study of ganetespib monotherapy in patients with genotypically defined advanced non-small cell lung cancer. Clin. Cancer Res. 19, 3068–3077 10.1158/1078-0432.CCR-12-3381 23553849 PMC3874465

[58] Queitsch C., Sangster T.A. and Lindquist S. (2002) Hsp90 as a capacitor of phenotypic variation. Nature 417, 618–624 10.1038/nature749 12050657

[59] Cowen L.E. and Lindquist S. (2005) Hsp90 potentiates the rapid evolution of new traits: drug resistance in diverse fungi. Science 309, 2185–2189 10.1126/science.1118370 16195452

[60] Rutherford S.L. and Lindquist S. (1998) Hsp90 as a capacitor for morphological evolution. Nature 396, 336–342 10.1038/24550 9845070

[61] Waddington C.H. (1942) Canalization of development and the inheritance of acquired characters. Nature 150, 563–565 10.1038/150563a0

[62] Flatt T. (2005) The evolutionary genetics of canalization. Q. Rev. Biol. 80, 287–316 10.1086/432265 16250465

[63] Jarosz D.F., Taipale M. and Lindquist S. (2010) Protein homeostasis and the phenotypic manifestation of genetic diversity: principles and mechanisms. Annu. Rev. Genet. 44, 189–216 10.1146/annurev.genet.40.110405.090412 21047258

[64] Jarosz D.F. and Lindquist S. (2010) Hsp90 and environmental stress transform the adaptive value of natural genetic variation. Science 330, 1820–1824 10.1126/science.1195487 21205668 PMC3260023

[65] Vincent B.M., Lancaster A.K., Scherz-Shouval R., Whitesell L. and Lindquist S. (2013) Fitness trade-offs restrict the evolution of resistance to amphotericin B. PLoS Biol. 11, e1001692 10.1371/journal.pbio.1001692 24204207 PMC3812114

[66] Cowen L.E., Singh S.D., Kohler J.R., Collins C., Zaas A.K., Schell W.A. (2009) Harnessing Hsp90 function as a powerful, broadly effective therapeutic strategy for fungal infectious disease. Proc. Natl. Acad. Sci. U. S. A. 106, 2818–2823 10.1073/pnas.0813394106 19196973 PMC2650349

[67] Whitesell L., Santagata S., Mendillo M.L., Lin N.U., Proia D.A. and Lindquist S. (2014) HSP90 empowers evolution of resistance to hormonal therapy in human breast cancer models. Proc. Natl. Acad. Sci. U. S. A. 111, 18297–18302 10.1073/pnas.1421323111 25489079 PMC4280614

[68] Azad A. K.M., Lawen A. and Keith J.M. (2017) Bayesian model of signal rewiring reveals mechanisms of gene dysregulation in acquired drug resistance in breast cancer. PLoS One 12, e0173331 10.1371/journal.pone.0173331 28288164 PMC5348014

[69] Jerby-Arnon L., Pfetzer N., Waldman Y.Y., McGarry L., James D., Shanks E. (2014) Predicting cancer-specific vulnerability via data-driven detection of synthetic lethality. Cell 158, 1199–1209 10.1016/j.cell.2014.07.027 25171417

[70] Sequist L.V, Waltman B.A., Dias-Santagata D., Digumarthy S., Turke A.B., Fidias P. (2011) Genotypic and histological evolution of lung cancers acquiring resistance to EGFR inhibitors. Sci. Transl. Med. 3, 90ra59 10.1126/scitranslmed.3002003 21430269 PMC3132801

[71] Zhao B., Pritchard J.R., Lauffenburger D.A. and Hemann M.T. (2014) Addressing genetic tumor heterogeneity through computationally predictive combination therapy. Cancer Discov. 4, 166–174 10.1158/2159-8290.CD-13-0465 24318931 PMC3975231

[72] Ryall K.A., Shin J., Yoo M., Hinz T.K., Kim J., Kang J. (2015) Identifying kinase dependency in cancer cells by integrating high-throughput drug screening and kinase inhibition data. Bioinformatics 31, 3799–3806 26206305 10.1093/bioinformatics/btv427 PMC4675831

[73] Kim J., Yoo M., Kang J. and Tan A.C. (2013) K-Map: connecting kinases with therapeutics for drug repurposing and development. Hum. Genomics 7, 20 10.1186/1479-7364-7-20 24060470 PMC3868238

[74] Ryall K.A., Kim J., Klauck P.J., Shin J., Yoo M., Ionkina A. (2015) An integrated bioinformatics analysis to dissect kinase dependency in triple negative breast cancer. BMC Genomics 16, S2 10.1186/1471-2164-16-S12-S2 26681397 PMC4682411

[75] Zhao B., Hemann M.T. and Lauffenburger D.A. (2016) Modeling Tumor Clonal Evolution for Drug Combinations Design. Trends in Cancer 2, 144–158 10.1016/j.trecan.2016.02.001 28435907 PMC5400294

[76] Jonsson V.D., Blakely C.M., Lin L., Asthana S., Matni N., Olivas V. (2017) Novel computational method for predicting polytherapy switching strategies to overcome tumor heterogeneity and evolution. Sci. Rep. 7, 44206 10.1038/srep44206 28287179 PMC5347024

[77] Wagle N., Grabiner B.C., Van Allen E.M., Hodis E., Jacobus S., Supko J.G. (2014) Activating mTOR mutations in a patient with an extraordinary response on a phase I trial of everolimus and pazopanib. Cancer Discov. 4, 546–553 10.1158/2159-8290.CD-13-0353 24625776 PMC4122326

[78] Al-Ahmadie H., Iyer G., Hohl M., Asthana S., Inagaki A., Schultz N. (2014) Synthetic lethality in ATM-deficient RAD50-mutant tumors underlies outlier response to cancer therapy. Cancer Discov. 4, 1014–1021 10.1158/2159-8290.CD-14-0380 24934408 PMC4155059

[79] Iyer G., Hanrahan A.J., Milowsky M.I., Al-Ahmadie H., Scott S.N., Janakiraman M. (2012) Genome Sequencing Identifies a Basis for Everolimus Sensitivity. Science 338, 221–221 10.1126/science.1226344 22923433 PMC3633467

[80] Takebe N., McShane L. and Conley B. (2015) Exceptional responders—discovering predictive biomarkers. Nat. Rev. Clin. Oncol. 12, 132–134 10.1038/nrclinonc.2015.19 25687910

[81] Casali P.G., Zalcberg J., Le Cesne A., Reichardt P., Blay J.-Y., Lindner L.H. (2017) Ten-Year Progression-Free and Overall Survival in Patients With Unresectable or Metastatic GI Stromal Tumors: Long-Term Analysis of the European Organisation for Research and Treatment of Cancer, Italian Sarcoma Group, and Australasian Gastrointestinal Tr. J. Clin. Oncol. 35, 1713–1720 10.1200/JCO.2016.71.0228 28362562

[82] Lipinski K.A., Barber L.J., Davies M.N., Ashenden M., Sottoriva A. and Gerlinger M. (2016) Cancer Evolution and the Limits of Predictability in Precision Cancer Medicine. Trends in Cancer 2, 49–63 10.1016/j.trecan.2015.11.003 26949746 PMC4756277

[83] Zhao B., Hemann M.T. and Lauffenburger D.A. (2016) Modeling Tumor Clonal Evolution for Drug Combinations Design. Trends in Cancer 2, 144–158 10.1016/j.trecan.2016.02.001 28435907 PMC5400294

[84] Thorlund K., Golchi S. and Mills E. (2017) Bayesian adaptive clinical trials of combination treatments. Contemp. Clin. Trials Commun. 8, 227–233 10.1016/j.conctc.2017.11.001 29696213 PMC5898557

[85] Zang Y. and Lee J.J. (2014) Adaptive clinical trial designs in oncology. Chin. Clin. Oncol. 3, 49 10.3978/j.issn.2304-3865.2014.06.04 PMC4369921 25811018

[86] Vyse S., Howitt A. and Huang P.H. (2017) Exploiting Synthetic Lethality and Network Biology to Overcome EGFR Inhibitor Resistance in Lung Cancer. J. Mol. Biol. 429, 1767–1786 10.1016/j.jmb.2017.04.018 28478283 PMC6175049

[87] Doebele R.C., Pilling A.B., Aisner D.L., Kutateladze T.G., Le A.T., Weickhardt A.J. (2012) Mechanisms of resistance to crizotinib in patients with ALK gene rearranged non-small cell lung cancer. Clin. Cancer Res. 18, 1472–1482 10.1158/1078-0432.CCR-11-2906 22235099 PMC3311875

[88] Berns K., Horlings H.M., Hennessy B.T., Madiredjo M., Hijmans E.M., Beelen K. (2007) A Functional Genetic Approach Identifies the PI3K Pathway as a Major Determinant of Trastuzumab Resistance in Breast Cancer. Cancer Cell 12, 395–402 10.1016/j.ccr.2007.08.030 17936563

